# High Oxygen Packaging of Atlantic Cod Fillets Inhibits Known Spoilage Organisms, but Sensory Quality Is Not Improved Due to the Growth of *Carnobacterium/Carnobacteriaceae*

**DOI:** 10.3390/foods10081754

**Published:** 2021-07-29

**Authors:** Anlaug Ådland Hansen, Solveig Langsrud, Ingunn Berget, Mari Øvrum Gaarder, Birgitte Moen

**Affiliations:** Nofima—Norwegian Institute of Food, Fisheries and Aquaculture Research, P.O. Box 210, N-1431 Ås, Norway; solveig.langsrud@nofima.no (S.L.); ingunn.berget@nofima.no (I.B.); Mari.Gaarder@Nofima.no (M.Ø.G.); birgitte.moen@nofima.no (B.M.)

**Keywords:** microbiota, bacteriota, spoilage bacteria, *Photobacterium*, *Carnobacterium*, sensory analysis, volatile components, MAP, vacuum packaging, freezing

## Abstract

Improved quality control and prolonged shelf life are important actions in preventing food waste. To get an overview of the bacterial diversity of fillets from live stored mature Atlantic cod, bacterial isolates were identified before and after storage (air and vacuum) and freezing/thawing. Based on the load of dominating bacteria, the effect of different packaging methods and a short freezing/thawing process on prolonged shelf-life was evaluated (total viable counts, bacteriota, sensory attributes, and volatile components). Hand filleted (strict hygiene) cod fillets had a low initial bacterial load dominated by the spoilage organism *Photobacterium*, whereas industrially produced fillets had higher bacterial loads and diversity (*Pseudomonas*, *Arthrobacter*, *Psychrobacter*, *Shewanella*). The identified bacteria after storage in vacuum or air were similar to the initially identified bacteria. Bacteriota analysis showed that a short time freezing/thawing process reduced *Photobacterium* while modified atmosphere packaging (MAP; 60% CO_2_/40% O_2_ or 60% CO_2_/40% N_2_) inhibited the growth of important spoilage bacteria (*Photobacterium,* *Shewanella, Pseudomonas*) and allowed the growth of *Carnobacterium*/*Carnobacteriaceae* and *Acinetobacter.* Despite being dominated by *Photobacterium,* fresh fillets stored in MAP 60% CO_2_/40% N_2_ demonstrated better sensory quality after 13 days of storage than fillets stored in MAP 60% CO_2_/40% O_2_ (dominated by *Carnobacterium/Carnobacteriaceae*). *Carnobacterium* spp. or other members of *Carnobacteriaceae* may therefore be potential spoilage organisms in cod when other spoilage bacteria are reduced or inhibited.

## 1. Introduction

One of the causes of food waste at the retail level is linked to a limited shelf life, and this is especially true for food with a relatively short shelf life such as fresh fish products [1,2,3]. It is also reported that fish has a high food loss in the EU (51%), of both edible and not fully utilized inedible parts of the fish [4]. In a circular economy, it is important to improve quality control and define best practices for a better utilization of fish raw materials. Atlantic cod (*Gadus morhua* L.) is an important species for the seafood industry connected to the Atlantic Ocean, and an improved knowledge of factors affecting shelf life is therefore important.

The quality deterioration of fish is primarily caused by bacterial growth and metabolism, autolytic changes, and chemical oxidation [5]. Such changes can lead to undesired development of volatile organic compounds (VOCs), resulting in off-flavors and off-odors. One of the most common VOC in cod is the pungent trimethylamine (TMA) caused by the reduction of trimethylamine oxide (TMAO) by specific spoilage bacteria such as *Photobacterium* [6,7]. There are, however, several different bacteria that can contribute to the fish spoilage through production of off-flavors and off-odors. Storage conditions such as temperature and packaging methods affect the bacterial composition, the lag phase and growth and hence also the presence of off-flavors and off-odors [7,8,9,10,11,12]. Although the fish muscle is considered sterile at the time of slaughtering or catch, bacteria from the skin, gastrointestinal tract, water, and processing environment can contaminate the fillets during processing [13]. *Photobacterium* spp. are the most dominant species in the intestinal microbiome of Atlantic cod and may contaminate the flesh during processing [14]. As far as we know, the bacteriota (bacterial composition) on cod processing machines has not been studied. However, in salmon processing plants, spoilage bacteria, such as *Pseudomonas* and *Shewanella*, were isolated from processing equipment after sanitation and *Photobacterium* were found in seawater and live fish, thereby enabling the cross contamination of fillets [15]. 

To extend shelf life, the processing and storage conditions should be optimized to prevent the growth of spoilage organisms present in fish. A common packaging method of gutted cod and cod fillets is aerobic storage in expanded polystyrene (EPS) boxes supplemented with ice to ensure a low chilling temperature [16]. Single packaged cod fillets and fillet products are also sold vacuum packaged or modified atmosphere packaged (MAP). Packaging with use of MAP is reported to preserve quality better than by vacuum packaging [6,11,12,17], and vacuum packaging is regarded as better to ensure quality and shelf life than aerobic storage [17,18]. However, a sufficient partial pressure of CO_2_ or headspace volume is required to obtain delayed bacterial growth compared to vacuum packaging [12,19,20,21]. 

A common gas mixture of MAP for fish fillets consists of carbon dioxide and nitrogen to suppress the growth of aerobic bacteria and bacteria sensitive to CO_2_, such as *Pseudomonas* [13]. The bacteriota at the end of the shelf life is reported to be dominated by *Photobacterium phosphoreum* which is relatively resistant to CO_2_ [12,22,23], or a mixed bacteriota (*Pseudomonas*, *Shewanella*, and *Photobacterium*) though under different storage conditions [24,25]. Some food packaging also include high levels of O_2_, which has shown to reduce the formation of TMAO to TMA in fish [23,24,26,27,28]. However, the bacteriota under high O_2_ storage has only been described in a few studies [16,23,24]. Studies also show that a mixture of bacteria presented during storage with a dominance of *Photobacterium* and *Psychrobacter*, followed by *Moritella*, *Carnobacterium*, *Shewanella*, and *Vibrio* as potential spoilage bacteria [29], and *Carnobacterium* spp. and *Vagococcus* spp. as part of the spoilage bacteriota of MAP sea bream [30].

A first indication of freezing as a technique to reduce luminous bacteria in fish came as early as in 1934 [31] and more recently studies showed that *P. phosphoreum* was not detectable in cod that had been stored frozen (6–12 weeks, −20 °C) and further refrigerated stored under modified atmosphere [22,32,33]. This indicated that a combination of a long-term freezing process to reduce *P. phosphoreum* and packaging with CO_2_ to inhibit *Shewanella* and *Pseudomonas* could extend the shelf life at refrigerated storage.

Even though many packaging studies of cod fillets have been performed, there is a lack of a full sensory descriptive analysis of the products, except for a description of heated samples [32,34]. Most of the studies where sensory analyses were included focused on overall freshness using the Quality Index Method (QIM) on raw whole fish [35], or evaluated the samples based on acceptance or rejection [6,36,37], or focussed on other species than cod [38]. 

Since total bacterial count does not necessarily correlate to spoilage due to the description of specific spoilage organisms (SSO) [13,39], it is important to consider the bacterial composition when deciding the shelf life and not only refer to the total viable count [29]. In order to better understand the diversity of spoilage bacterial communities, culture-independent analyses can provide additional information compared to traditional cultivation based methods [40]. Improved knowledge about how the bacteriota and sensory quality will respond to different packaging atmospheres and freezing strategies is therefore crucial, and it is an important step towards the reduction of food waste. 

The aim of the present study was therefore to identify packaging concepts (different atmospheres combined with or without a freezing/thawing process) that can direct the bacteriota of cod fillets towards a composition with fewer detrimental sensory effects, thus increasing the shelf life. To obtain this, we identified bacteria on fillets from different processing plants (initially and after storage and a long-term freezing process) and investigated the effect of different packaging atmospheres on a range of potential spoilage organisms from raw materials and stored cod fillets. Furthermore, the effect of packaging atmospheres and a short-term freezing on the microbial growth, bacteriota, sensory attributes, and volatile compounds in cod filets during refrigerated storage were determined. 

## 2. Materials and Methods

**Figure 1:**
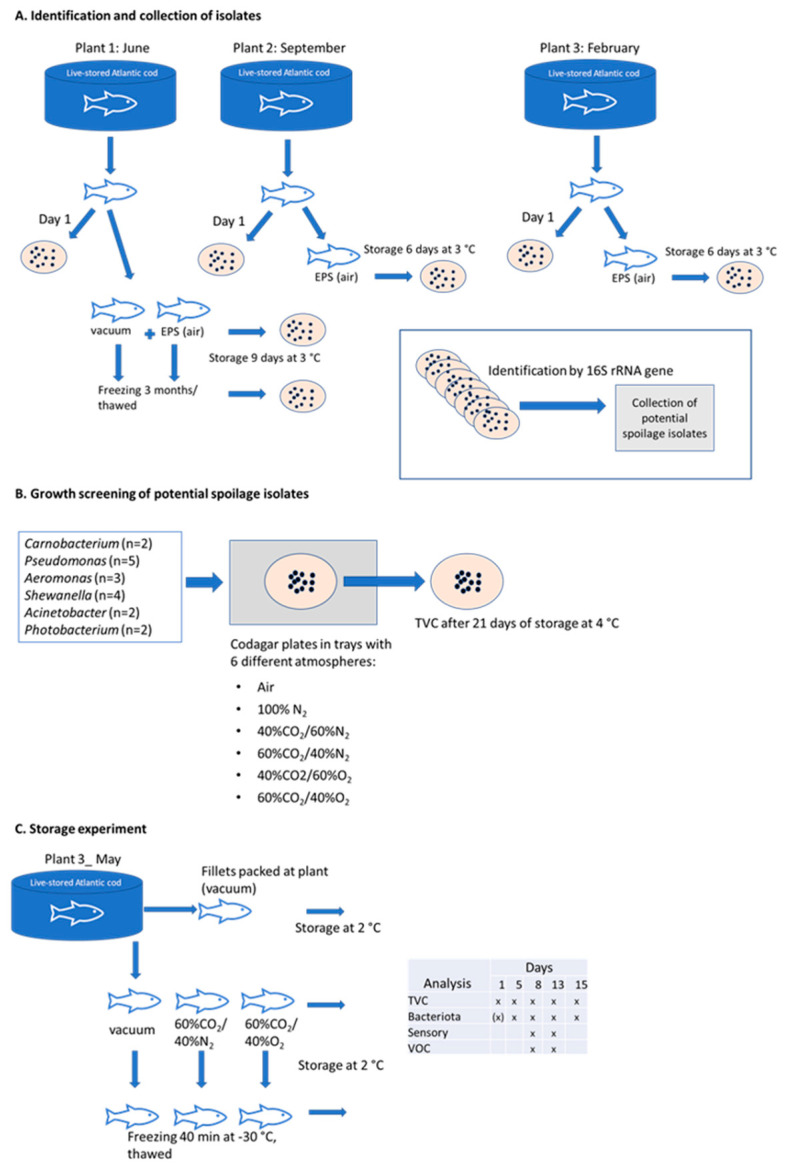
An overview of the three different parts (**A**–**C**) of the presented study. (**A**): Identification and collection of isolates. Cod fillets from three different plants were collected and stored at 3 °C in EPS boxes (air) for 6 (Plant 2 and 3) or 9 days (Plant 1). Fillets from Plant 1 were also frozen/thawed and stored in EPS boxes (air) and vacuum packaged for 9 days. Bacterial colonies were collected from agar plates at arrival (day 1) or after storage. The bacterial colonies were identified by partial 16S rRNA gene sequencing. (**B**): Growth screening of potential spoilage isolates. The growth of selected isolates from part (**A**) at 6 different atmospheres was tested on cod agar in trays. The total viable counts were registered after 21 days at 4 °C. (**C**): Storage experiment. Fresh fillets from Plant 3 were vacuum packaged (from commercial production) and packaged specific designed for the study by use of vacuum, and modified atmosphere (MAP) containing 60% CO_2_/40% N_2_ and 60% CO_2_/40% O_2_, all stored at 2 °C for up to 15 days. In addition, half of the packages were subjected to a short freezing process (40 min at −30 °C) before thawed and stored the same way as for the fresh fillets. Samples for TVC and bacteriota were collected after 1, 5, 8, 13 and 15 days, while samples for sensory- and volatile component (VOC) analysis were collected after 8 and 13 days. All fish samples used were of wild matured cod stored live in net pens for about two to four weeks prior to slaughtering (mean weight 5 kg) and pre-rigor filleting.

The presented work contains three parts: (1) Identification of bacterial strains from cod fillets (initial bacteriota and during a pre-storage test), (2) growth screening of selected bacterial strains on cod agar medium trays packaged with different gas mixtures, and (3) a storage experiment with packaging and short-term freezing. Figure 1 shows an overview of these three different parts.

### 2.1. Characterization of Bacterial Strains from Cod Fillet

#### 2.1.1. Fish Material

Cod fillets (*Gadus morhua* L.) were transported from three different processing plants in Norway within a period of one year, resulting in three different batches. Batch 1 was delivered in June (2014) and originated from a research station with a fish farm site (Plant 1). Batches 2 and 3 were delivered in September (2015) and February (2016), respectively, from two different commercial processing plants for fillet production (Plant 2 and 3, respectively). All samples were Atlantic cod (wild matured cod) stored live without feed supply in net pens for about two to four weeks prior to slaughtering (mean weight 5 kg). The current Norwegian regulations state that wild caught cod can be live stored in sea cages up to 12 weeks, and during the first four weeks feeding is not required [41], and the quality of fish stored live in net pens for about four weeks is reported to be unchanged compared to the initial quality [42]. Live storage of cod is a method to extend the fishing season and is used as a supplement during periods with bad weather conditions. The pre-rigor filleting (by hand at Plant 1) and packaging were performed at site the same day as slaughtering, and the fillets were packaged in EPS boxes wrapped with plastic sheets of polyethylene (OTR 5000 cm^3^ O_2_/m^2^/day) and added wet ice on top. The boxes were transported to the laboratory at Nofima (Ås, Norway) by airplane and truck (arrival the day after) for sampling at arrival (Day 1). 

To map a broad range of microbes that can be present on cod after filleting and storage, the bacteriota on cod fillets one day after processing and after storage under different conditions relevant for the cod production chain was investigated. 

Half of the fillets from Plant 1 were packaged at Day 1 into vacuum prior to further storage, and half continued stored in EPS box. Furthermore, half of the EPS-samples and the vacuum samples were frozen at Day 1 for 3 months (−20 °C), as a pre-test of long-term frozen stored fillets, then thawed overnight at 1 °C before further storage. Both the thawed and the fresh samples from Plant 1 were sampled after 9 days of refrigerated storage (*n* = 6) (mean storage temperature 3 °C). The fillets from Plant 2 and 3 were sampled (*n* = 5) after 6 days of aerobic storage (mean storage temperature 3 °C). The 6 and 9 days were empirically chosen to achieve approximately similar levels of total bacterial count related to different initial count levels (Table 1), and at this time of storage, to obtain bacteriota that can be important for quality and shelf life. 

#### 2.1.2. Microbial Sampling

Microbial samples were taken from the fillets (skin and meat, separately) of Plant 1 and analyzed at Day 1 (*n* = 6) by use of sterile swabs (Cotton Tipped Applicator, single tip, OneMed, Helsinki, Finland) of a 5 × 20 cm surface of the fillet, suspended in 5 mL Pepton water and grown on Long & Hammer medium [43] at 15 °C for 5–7 days. The Long & Hammer agar contained 1% NaCl. 

Additionally, bacterial plate count after 1 and 6/9 days of storage was performed by taking 3 × 3 cm and 1 cm depth (approximately 10 g) at the dorsal part of the fillets (without skin). The samples were homogenized in a stomacher for 60 s, and appropriate 10-fold dilutions were made and spread on Iron agar plates (Oxoid, Basingstoke, Hampshire, U.K.). Incubation was at 15 °C for about 5 days.

To identify the taxonomy of the cultivated bacteria from the three batches, 15 colonies (randomly selected from one sector per readable agar plate per fish fillet) for each sampling time were selected for DNA sequencing (16S rRNA gene).

#### 2.1.3. 16S rRNA Gene Sequencing of Colonies

Colonies were identified from agar plates by partial 16S rRNA gene sequencing (universal primers [44] covering variable regions 3 and 4 in the 16S rRNA gene (V3 and V4) directly on colonies using a variation of a yeast colony PCR method as described before [45,46]. Amplification was performed using 0.25 µmol/l (each) primer, 5 µL of 5 Prime HotMastermix (AH Diagnostics, Oslo, Norway) to a total volume of 25 µL. The cycling conditions were: 94 °C 2 min, then 30 cycles of denaturing at 94 °C for 30 s, annealing at 60 °C for 30 s, extension at 72 °C for 30 s, and a final extension at 72 °C for 7 min. The PCR products were purified before sequencing, using ExoSap-IT (USB Corp., Cleveland, OH, USA). Sequencing PCR was carried out using the Big Dye^®^ Terminator v1.1 Cycle Sequencing Kit (Applied Biosystems, Dublin, Ireland) according to the manufacturer’s instructions with the forward universal 16S rRNA gene primer. The sequencing reactions were carried out in 25 cycles of 96 °C for 15 s and 60 °C for 4 min. A BigDye XTerminator Purification Kit (Applied Biosystems) was used according to the manufacturer’s recommendations to clean up the sequencing reactions and sequencing was performed on an ABI PRISM 3130xl Genetic Analyzer (Applied Biosystems). The taxonomy was identified using the RDP (Ribosomal Database Project) SeqMatch (http://rdp.cme.msu.edu/, accessed on 25 June 2014, 21 October 2015, 16 March 2016). The thresholds used in the RDP search were as follows: both type and non-type strains, both uncultured and isolates, only good sequences >1200 nt and KNN = 1. 

#### 2.1.4. Muscle pH

The pH was measured in triplicate directly in the cod fillets using a Knick pH meter (Knick GmbH & Co, Berlin, Germany) and muscle electrode S/N 5290739 (Mettler Toledo, Urdorf, Switzerland), only from Plant 1, at Day 1 and Day 6/9.

### 2.2. Growth Screening of Selected Bacteria Strains on Cod Agar Medium Trays 

Based on abundance and knowledge of spoilage potential [13,15,24,40], 20 bacterial strains from the identified bacterial isolates and from culture collection, were selected for screening of growth (Table A1). Selections were performed to represent both bacteria that previous has been reported to cause spoilage, and bacteriota (initial and after storage) from the different processing plants. 

To make an effective screening of the selected bacterial strains, a model system of cod agar plates were made, packaged with different gas mixtures (Table A2) and incubated at 4 °C. Cod agar was prepared as described in an earlier study [47], by use of 5 kg of fresh Atlantic cod fillets bought from a retailer. 

The different bacterial cultures were incubated aerobically at 15 °C on Iron agar for about 5 days, colonies were picked and further cultivated in Long & Hammer broth (contained 1% NaCl) for 2 days at 15 °C, until reaching 8.1 ± 0.6 log10 CFU/mL. Each bacterial culture was diluted to achieve 5 log10 CFU/mL and an inoculate volume of 100 µL was used for the cod agar plates. There was one agar plate (without the lid) per packaged sample, and one agar plate per strain per gas mixture was included, which means 2–5 strains for each genus. 

The packaging consisted of APET preformed tray (with 50 mm drawing depth), Biaxer topweb (65 XX FP AFM, Wipak, Oy, Finland) using a tray sealer (Multivac T200, Wolfertschwenden, Germany) with different pre-mixtures of gas (Nippon Gases Norge AS, Oslo, Norway). 

After 13 days of storage, all packages were measured for the content of CO_2_ and O_2_ (Table A2) using a ChackMate 9900 O_2_/CO_2_ analyser (PBI Dansensor, Ringsted, Denmark). The samples were stored at 4 °C for 3 weeks. 

To analyze the bacterial counts, the cells were rinsed off the agar plates using one ml sterile peptone water and a L-shaped spreader (VWR International, Oslo, Norway). The viable cell counts analysis was performed using an automated spiral plater (WASP Whitley automatic WB 05 SH, VWR International) on Iron agar plates, incubated at 15 °C for 3–5 days (read by ProtoCOL 2, Synbiosis (Cambrigde, United Kingdom)). 

### 2.3. Storage Experiment of Pre-Rigor Processed Cod Loins 

Based on results from the initial experiments and existing literature [23,24,27,28,48], it was hypothesized that a combination of CO_2_ and O_2_ (high levels of both O_2_ and CO_2_) would show a greater inhibitory effect on bacterial growth than CO_2_ and N_2_ (with high levels of CO_2_) and that vacuum packaging would allow for rapid bacterial growth. Furthermore, it was hypothesized that a short-term freezing process would eliminate a fraction of the initial bacteriota (e.g., *Photobacterium*) and delay microbial growth further. To test the hypotheses, industrially produced cod loins processed at Plant 3 were packed with different atmospheres as described in Section 2.3.1 (“Vacuum”, “CO_2_” and “O_2_/CO_2_”) and with and without being subjected to a short-term freezing after packaging, followed by a thawing process/refrigerated storage. 

#### 2.3.1. Fish material and Packaging

Atlantic cod was caught at the coast of northern Norway and stored live in cage (feed-deprived) for about four weeks (Senjahopen, Norway) before slaughter. Filleting was performed within two hours after slaughtering (mean weight was 4.8 kg; headed and gutted). 

The fillets were cut into loins (dorsal part of the fillet) using a Valka Cutter water jet machine (Valka, Reykjavik, Iceland). Three different packaging methods were used: (1) MAP with 60% CO_2_ and 40% N_2_ and with a CO_2_-emitter pad (“CO_2_”), (2) MAP with 60% CO_2_ and 40% O_2_ (“O_2_/CO_2_”), and (3) vacuum packaging (“Vacuum”). In addition, an industrial processed sample of the ordinary production at the processing plant were used (line caught cod at the coast of northern Norway, delivered to the processing plant immediately after catch). The industrial processed samples (designated “Industry”) were filleted, cut into loins, and vacuum packed 2–3 h after processing of the other test samples. 

A thermoformer packaging machine (Multivac R145, Multivac, Germany) was used for modified atmosphere packaging (“CO_2_”) and vacuum packaging at the processing plant. Drawing depth of vacuum packages was 35 mm (package volume 400 mL) and 40 mm for MAP (627 mL volume, gas/product volume ratio of 1/1). Film used for the bottom web was NICE XX 18 Black (PE/PA/EVOH/PA/PE), thickness of flat material 350 µm (Wipak Oy, Nastola, Finland). For the top web SC XX 3PA (PA/PP/PA/EVOH/PA/PE) with thickness of flat material 80 µm (Wipak Oy) was used. For the “Industry” samples SC 4 PA (PA/PP/PA/PE) was used both for top and bottom web (thickness of flat material 90 µm, Wipak Oy). The industrial processing was part of the processing plants regular production process of frozen single packed fillets.

For safety reasons, the O_2_/CO_2_ samples were packed at Nofima (packaging machine, Multivac T200, adjusted to high levels of O_2_): the fillets were vacuum packaged at the commercial processing plant, transported in EPS Air boxes added ice, and hygienically repackaged into MAP (40% O_2_ and 60% CO_2_) the day after filleting. Preformed trays (50 mm depth) of HDPE (600 mL volume) and a Biaxer (PET/PE/EVOH/PE) top web were used, with similar gas/product volume ratio as for the “CO_2_”. The gas mixture for the “O_2_/CO_2_” was measured to be 39.2% O_2_ and 55.3% CO_2_ in an empty package at the day of packaging. 

In “CO_2_” packages, a CO_2_ emitter pad with 35 mL liquid absorption capacity and 230 mL CO_2_ emission capacity (XP-CO2-35-230-070175-Y, 70 × 175 mm,; Mc Airlaid’s GmbH, Steinfurt, Germany) was added to achieve an optimal CO_2_ availability. The CO_2_ emitter develop CO_2_ gas inside the package initiated by the liquid loss from the fish sample. 

Liquid absorbent pad with 110 mL absorption capacity (MGS-110-070175-70, 70 × 175 mm; Mc Airlaid’s GmbH), was used for the O_2_/CO_2_ and the Vacuum packages.

The CO_2_ and O_2_ were analyzed at each sampling time by a CheckMate 9900 O_2_/CO_2_ analyser (PBI Dansensor, Ringsted, Denmark). 

#### 2.3.2. Freezing 

Based on results from the pretest characterizing bacterial strains from fresh and thawed samples, half of the samples (CO_2_, O_2_/CO_2_ and Vacuum, not the Industry samples) were frozen by use of a Torry continuous air blast freezer (IQF; Individual Quick Freezing, Refrigeration Aberdeen LTD, Aberdeen, Scotland, UK) immediately after packaging. Freezing time was 40 min with air temperature monitored to be −30 °C. After freezing, the samples were placed in EPS boxes added wet ice for chill transport, in which the samples were thawed at arrival to the Nofima laboratory (Ås, Norway) the day after. 

In the following, thawed samples will be designated as “CO_2_-T”, “O_2_/CO_2_-T” and “Vacuum-T”. 

#### 2.3.3. Temperature and Sampling

The mean temperature for the thawed samples and the ordinary chilled samples during transport were −2.3 ± 0.9 °C and 0.8 ± 0.6 °C, respectively (temperature loggers used: Kooltrak GmbH, Kiedrich, The Netherlands) and it took about 24 h for the thawed samples to reach the same temperature as the chilled samples (2 °C). Sampling was performed after 1, 5, 8, 13 and 15 days (*n* = 3 per sampling and treatment) at 2 °C storage (loggers used: Ecolog TN4-L, Elpro-Buchs AG, Buchs, Switzerland) for bacterial analyses. Day 0 was the time of processing and packaging at the industrial processing plant. Volatile components and sensory analysis were performed on day 8 and 13, based on current time of shelf life.

#### 2.3.4. Culture-Dependent Analyses of Bacteria (Plate Count)

Analyses of total bacterial count were performed as earlier described (Section 2.1.2) of 3 × 3 cm cut from samples (Iron agar).

#### 2.3.5. Culture Independent Analysis of Bacteria

Forty-five ml of the stomacher solutions were centrifuged at 13,000× *g* for 5 min. The pellets were frozen at −20 °C until DNA extraction using the Fast DNA-96 Soil Microbe kit (MP Biomedical) and following the manufacture’s MP-96 Inhibitor Removal Plate protocol.

PCR was performed in triplicate and paired end sequencing (2 × 150 bp) was performed using the protocol presented in Caporaso et al. [49] and as described before [50]. The library quantification and sequencing were performed at Nofima. The MiSeq Control Software (MCS) version used was RTA v1.18.54. 

The forward and reverse reads were joined in QIIME version 1.9.1, and the barcodes corresponding to the reads that failed to assemble were removed. The sequences were then demultiplexed in QIIME allowing zero barcode errors and a quality score of 30 (Q30) using the QIIME toolkit [51]. The maximum and minimum number of sequences per sample was 171,601 and 64,940, respectively. Reads were assigned to their respective bacterial taxonomy using an openref Operational Taxonomic Unit (OTU) picking workflow. Reads that did not match a reference sequence were discarded resulting in 3779 OTUs with *n* > 2, each of these represents a phylotype and may be a representative of a bacterial species. The level 6 (genus) table derived from QIIME was used for further statistical analyses.

#### 2.3.6. Volatile Organic Compounds (VOCs)

Dynamic headspace/GC-MS analyses of volatile organic compounds (VOCs) were performed on samples from the packages stored for 8 and 13 days. Samples were cut from the same loin as performed for the bacterial analyses and the sensory analysis. The content of volatiles was analyzed by dynamic headspace/GC-MS as described by [23,52] with small modifications to the method. The peaks were integrated, and compounds tentatively identified with MSD Chemstation software (E.02.02.1431) and NIST/EPA/NIH Mass Spectral Library (version2.0 g, 2012). Concentrations of the individual volatiles were calculated as µg/g sample based on an internal standard.

#### 2.3.7. Sensory Analysis

To describe the objective perception of the various samples, a trained panel performed a Quality Descriptive Analysis (QDA), ISO 13299:2016 (E) of the samples [53]. The panel consisted of 10 subjects employed exclusively to work as sensory assessors at Nofima AS (Ås, Norway). Assessors are selected based on their sensory abilities and trained according to recommendations in ISO 8586-1:2012 (E) [54]. The sensory laboratory is designed according to guidelines in ISO 8589: 2007(E) [55] and electronic registration of data (EyeQuestion^®^, Logic8 BV, Utrecht, The Netherlands).

The assessors were trained and calibrated on two samples, fresh and stored cod, for the purpose to agree on the variation in attribute intensity. The samples were evaluated for the intensity of sensory odor attributes sour, seawater, cloying, fermented sour, yeast, chemical, sulfur, ammonia and pungent. Appearance was evaluated for the intensity of whiteness and glossiness (Table A3), evaluated after the odor attributes. In total, 21 loins were cut into ten pieces (one for each assessor) and served during five sessions with at least fifteen minutes break between each session. The coded samples (15 °C at time of evaluation) were served in blind trials randomized according to sample, assessor, and replicate. The samples were served to each assessor as raw samples in plastic cups with a three-digit code, with lid, with an approximate surface size of 3 × 3 cm (samples placed with skin side of the fillet towards the bottom of the cup). The intensity of each odor attribute was perceived by sniffing into the newly opened plastic cup. 

The panelists recorded their results at an individual rate on a 15-cm non-structured continuous scale with the left side of the scale corresponding to the lowest intensity, and the right side corresponding to the highest intensity. The software transformed the responses into numbers between 1 (low intensity) and 9 (high intensity).

#### 2.3.8. Statistical Analyses

Bacterial numbers were log10 transformed and mean values and standard error of the mean calculated in Minitab (Minitab 18.1, 2017, www.minitab.com, accessed on 29 May 2019). One-way ANOVA and Fisher least square of differences at 95% confidence were used to calculated differences between means. 

Logarithmic change of the selected bacteria strains on cod agar medium trays, was calculated by subtracting the initial number of bacteria from the number after incubation for each strain. Statistical tests were done using variances between strains to calculate error. 

Total counts (log10 transformed) were analysed by ANOVA using gas (CO_2_, O_2_/CO_2_, Vacuum), process (Fresh, Thawed), day, and second order interactions as effects. The industry sample was not included as a part of the ANOVA. Terms were considered significant for *p*-values below 0.05. Post hoc comparisons were done using contrasts for the expected marginal means, Tukey-HSD adjustment of *p*-values was performed for multiple testing adjustments. 

The effects of experimental factors (gas, processing, days of storage) and their interactions on bacteriota and volatile compounds were tested using fifty-fifty MANOVA [56] on each of the datasets separately. Rotation tests [57] were applied for the false discovery rate (FDR) adjustment of *p*-values (considered significantly different when *p*-values below 0.05). Log transformation of the data were performed to improve normality conditions. Because there are many zeros in the data for volatile compounds, an offset corresponding to 1% of the minimum value was added to the data prior to log transformation. Bacteriota was analysed at level 6, i.e., the genus level, and only genera with an average abundance above 0.01 or a maximum of 0.1 were included in the analyses, hence the focus was on the most abundant genera, twelve genera passed the filter (Table A4).

The sensory data were analysed using a linear mixed model comprising the factors product (all seven different packaging variants), replicate and assessor and the second order interactions. Assessor and interactions involving assessors were considered random whereas the other factors were fixed. 

Principal component analysis (PCA) was performed for each of the data sets from the experiment (bacteriota, sensory profiles and volatile compounds) to visualise and explore effects of the packaging concepts. 

Multivariate analyses (fifty-fifty-MANOVA and PCA) were done in Matlab (Mathworks, R2018b), whereas ANOVA on total counts were done using R [58], and the sensory analyses were done with EyeOpenR (EyeQuestion Software 4.4.6, Logic8 BV, The Netherlands). 

## 3. Results and Discussion

### 3.1. Bacterial Levels and Bacteriota in Cod Fillets from Three Different Producers before and after Storage at Different Conditions

#### 3.1.1. Bacterial Levels and Diversity on Fillets One Day after Slaughter

Fresh cod fillets, processed immediately after slaughtering, were obtained from three different producers at different time points. All samples were of live stored cod (feed- deprived) that had previously been shown to keep stable fillet quality during the first four weeks of storage [42]. The pH of the fillets from Plant 1 was 6.9 ± 0.2 at Day 1, and 6.5 ± 0.3 and 6.6 ± 0.3 for the air and vacuum stored samples respectively (similar for fresh and thawed samples), which is in accordance with previous results on longline caught cod and farmed cod [23,59].

Large variations in the counts and the bacteriota on cod fillets one day after slaughter were found and reflected most likely differences in contamination during processing. Fillets from Plant 1, which were hand filleted under strict hygiene conditions, had a very low initial bacterial number with a clear dominance of *Photobacterium* (93% of the isolates from fillets and 79% from skin samples) (Table 1). To our knowledge bacteriota on fillet products of live stored wild Atlantic cod (northeast arctic cod) is not previously described. However, a similar bacteriota has been described for hand filleted, farmed Atlantic cod [60]. A dominance of *Photobacterium* is also typically found on the fillets of wild caught cod raw materials and dominating in the intestinal microbiome [14,37,61,62]. In Plant 1, the total viable count numbers were lower on skin compared to previously reported (3–6 log10 CFU/g) [63]. 

Industrially produced fillets from Plant 2 had the highest number of bacteria among the processing plants, including *Pseudomonas* (28%), *Acinetobacter* (22%), and *Shewanella* (11%), but no *Photobacterium* were detected. Plant 3 had also high total bacterial counts (1000 times higher than Plant 1) and the most dominant bacteria belonged to *Pseudomonas* (30%), *Arthrobacter* (26%), and *Psychrobacter* (19%). Although they represented only a small fraction (3.8%) of the total bacteriota, the level of *Photobacterium* on Plant 3 fillets was higher than for Plant 1 fillets. The fillets from Plant 2 and 3 contained higher bacterial numbers and the bacteriota was typical for what is found in the seafood processing environments [13,15,64]. A variation between batches was found, as shown in Table 1. Differences between lots and fish species are also reported by Zotta et al. [65]. In contrast, the bacteriota on fillets from Plant 1 (hand filleted) was more typical for the fish raw material [15]. Although the total number of colonies identified were 176, we cannot rule out that the diversity of the initial bacteriota could be higher given more colonies. However, it is likely that the initial bacteriota on cod industrially processed fillets is a combination of the bacteriota of the raw material and bacteria contaminating the fillets during processing.

#### 3.1.2. Effect of Anaerobic and Aerobic Storage Conditions on the Bacterial Level and Bacteriota

Interestingly, fillets stored aerobically, or vacuum packed to a bacterial count of about 6 log10 CFU/g showed similar bacteriota as the initial bacteriota of the fillets (the fresh samples, Table 2). *Photobacterium* dominated in hand fileted cod from Plant 1 (92% and 98%, aerobically and vacuum packaged samples, respectively, from initially 93%) and was absent (<4.3% based on the number of isolates analyzed) in Plant 2 fillets, which was still dominated by *Pseudomonas* (39% from initially 28%), *Shewanella* (30% from initially 11%), and *Acinetobacter* (17% from initially 22%). In fillets from Plant 3, *Pseudomonas* still dominated, as also reported on vacuum packaged cod fillets [23], but the *Photobacterium* fraction had increased to 24% (initially 3%). Earlier reports have described that vacuum packaging of fresh fish can inhibit *Pseudomonas*, but it does not necessarily increase shelf life as *Photobacterium* or *Shewanella* (depending on initial numbers) will spoil the product [66,67,68]. Still, vacuum packaging is a common technology for fresh cod consumer packages, but the results of both the present and earlier studies show that it has no improved effect on the microbial shelf life compared to aerobic storage.

Most studies report bacterial content or bacteriota after a time of storage, and only a few studies refer to initial detection of bacteriota, like Hovda et al. [60] and Antunes-Rohling et al. [29]. They found *Pseudomonas*, *Photobacterium* and *Shewanella* both at Day 0 and after storage for hand filleted cod, similar as shown in the present study. 

As expected, the storage time needed to reach bacterial levels associated with spoilage was longer when the initial contamination number was low. Thus, the shelf life of cod fillets stored aerobically and in vacuum seemed to be directly determined by the initial level and composition of the bacteriota. 

#### 3.1.3. The Effect of Freezing-Thawing and Vacuum Packaging on the Bacterial Level and Bacteriota

The combination of vacuum packaging and freezing could theoretically improve the microbial shelf life, combining the inhibition of *Pseudomonas* and lethal effect on *Photobacterium* after thawing. In the present study, long-term frozen storage (3 months at −20 °C) reduced the total bacterial count of the thawed, refrigerated storage fillets and eliminated *Photobacterium* of Plant 1 fillets. A lethal effect of long-term freezing on *Photobacterium* has previously been reported by others [22,32,33,69]. In addition, it allowed the growth of other bacteria, dominated by *Pseudomonas*, for both the thawed vacuum packaged fillets and the aerobically stored thawed fillets (85% and 98%, respectively) (Table 2). 

### 3.2. Growth of Potential Spoilage Bacteria in a Cod Agar Model System under Different Atmospheres

Several studies have suggested that packaging with carbon dioxide gas combined with oxygen can extend the shelf life of cod [6,16,23,24,27,28,70]. Since the initial bacteriota varied considerably between processing plants, we screened a selection of potential spoilage bacteria isolated from cod fillets stored under different packaging atmospheres to investigate how different combinations of CO_2_, O_2_, and N_2_ inhibit growth (Table 3). Although we observed some strain differences within genera, we found that a combination of high concentrations of CO_2_ (60%) and O_2_ (40%) gas completely inhibited growth of *Photobacterium*, *Shewanella*, *Aeromonas,* and *Acinetobacter*, and partially *Pseudomonas* when grown on cod juice agar plates (Table 3). In general, the highest reduction or growth inhibition compared to aerobic storage was found for 60% CO_2_/40% O_2_, followed by 40% CO_2_/60% O_2_, 60% CO_2_/40% N_2_ and 40% CO_2_/60% N_2_. This corresponds to results obtained on *Shewanella* [71] and *Photobacterium* [72] showing highest inhibition of bacterial growth by the combination of CO_2_ and O_2_. The additive effect of oxygen together with CO_2_ on growth reduction was most notable for *Photobacterium*, *Shewanella*, and *Aeromonas*. Among the genera tested, *Carnobacterium* appeared to have highest tolerance to the different combinations of gas tested, including CO_2_ and O_2_. 

Restricted oxygen conditions (100% N_2_, 0.02 ± 0.0% O_2_, Table A2) had an overall insignificant effect on the bacterial growth compared to aerobic storage (Table 3). This result supported the findings in the storage tests described above (Section 3.1.3), that virtually all specific spoilage bacteria in cod may grow relatively fast under oxygen restriction (with no CO_2_), and the final dominating genera may be dependent on the initial contamination and probably also storage temperature.

Immediately after packaging, the gas mixtures were measured to be 37.7% CO_2_, 60.0% O_2_ and 57.9% CO_2_, 40.1% O_2_ for the 40% CO_2_/60% O_2_ and the 60% CO_2_/40% O_2_ mixtures, respectively (measurements only on two of the gas mixtures), showing good compliance to the pre-mixture of the packaging gases. 

### 3.3. Storage Experiment of Pre-Rigor Processed Cod Fillets

Based on the mapping of microbial levels before and after storage of cod under different conditions and the growth potential of bacteria associated with cod, we designed an experiment combining both freezing/thawing and the MAP conditions that showed the most promising results in the growth screening to measure the effect of TVC, bacteriota, VOCs and sensory quality on industrial processed cod fillets. A short-time freezing (40 min at −30 °C) was chosen instead of the previous long-term freezing process as a bacteria inhibitory treatment after the packaging, allowing short-time occupying of freezing capacity at the processing plant prior transport to the market. Vacuum was also tested as this is a commonly used packaging method of cod fillets in Norway. In addition, vacuum packaged fillets of traditional wild catch of Atlantic cod were included as a reference (called “Industry”).

#### 3.3.1. The Effect of Packaging Atmosphere on Development of the Bacteriota during Storage

**Figure 2:**
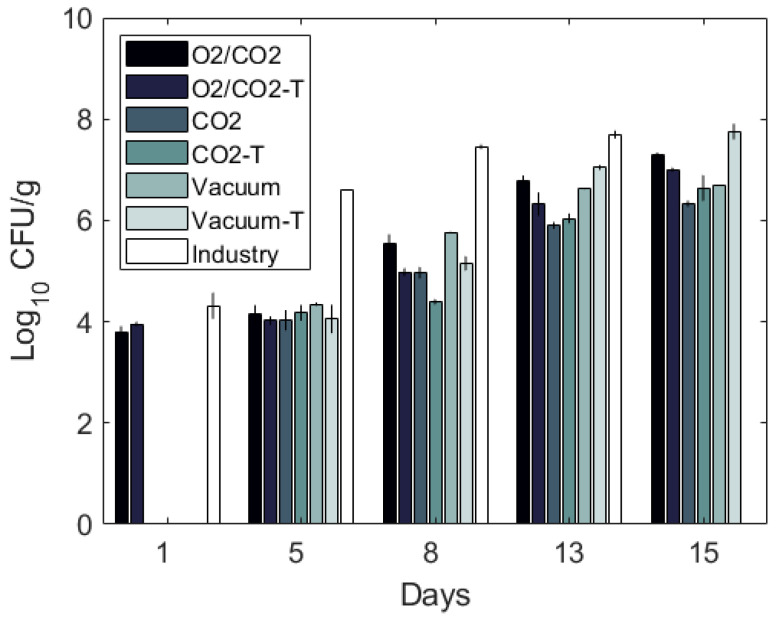
Total bacterial count (log10 CFU/g) (mean ± standard error of the mean, SEM) per packaging method of cod loins during storage (2 °C). The “CO_2_” samples are packaged with 60% CO_2_, 40% N_2_ and a CO_2_ emitter, and “O_2_/CO_2_” is packaged with 60% CO_2_ and 40% O_2_. “Industry” samples and “Vacuum” samples are packaged with vacuum. The samples marked with “T” are thawed samples, and the “CO_2_”, “O_2_/CO_2_”, “Vacuum” and “Industry” are fresh packaged fillets.

Variations in the bacterial growth rates were found between packaging atmospheres for fresh cod fillets during storage (Figure 2). The lowest level of bacteria after 15 days of storage was found in cod packed with 60% CO_2_ and highest in cod packed with O_2_/CO_2_. 

The content of CO_2_ in package headspace during storage can partial explain this. It was measured to be 53.0 ± 1.5% and 35.3 ± 3.2 for the 60% CO_2_/40% N_2_ and 60% CO_2_/40% O_2_ mixtures, respectively (no differences between fresh and thawed) and 59.7 ± 2.3% O_2_ during storage for the fresh and thawed O_2_/CO_2_ packages. 

**Figure 3:**
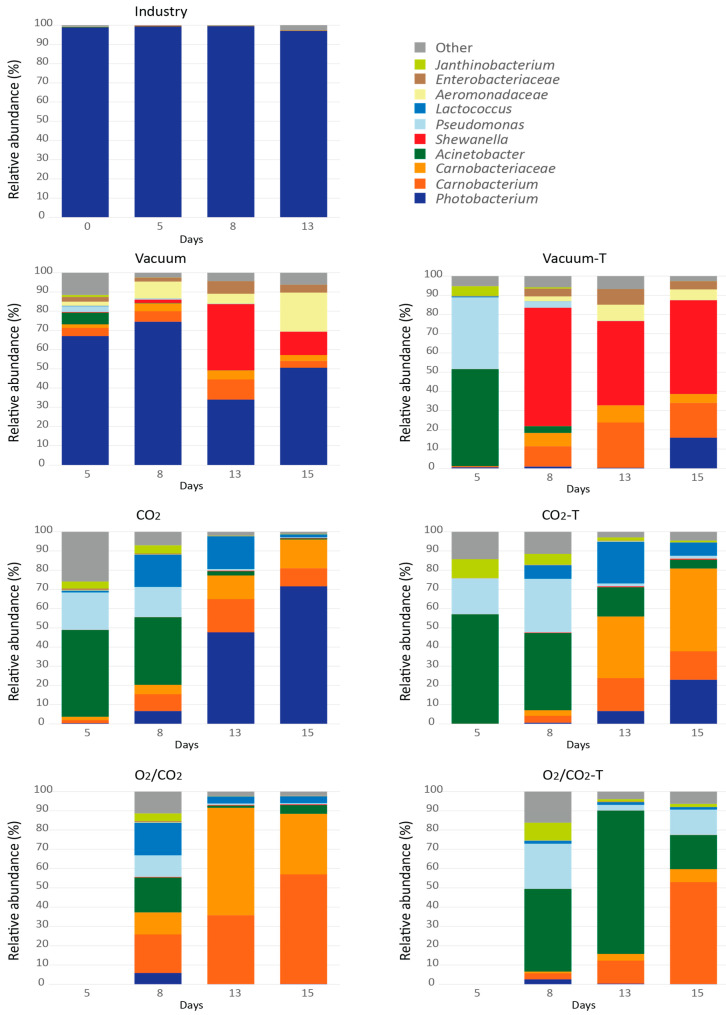
The dominating bacteriota (relative abundance) during storage with different packaging and processing. Each point is represented as an average of one to three parallels. Taxa with abundance above 1% across all samples is represented, the rest is presented as “Other”. Taxa is represented at either genus (g) or family (f) level by different colors: 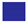 *Photobacterium*; 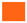 *Carnobacterium*; 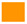 *Carnobacteriaceae*; 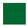 *Acinetobacter*; 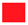 *Shewanella*; 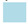 *Pseudomonas*; 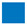 *Lactococcus*; 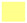 *Aeromonadaceae*; 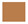 *Enterobacteriaceae*; 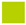 *Jantinobacterium*; 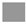 Other. Day 5 samples from O_2_/CO_2_ are missing from the MiSeq analysis and are marked with grey shading in the figure.

Profound differences were also found for the bacteriota between the packaging atmospheres (Supplementary Table S1 and Figure 2 and Figure 3). A rapid growth of *Photobacterium* was observed in vacuum packaged samples. After 13 days, the total growth plateaued to some degree, and the bacteriota was dominated by *Photobacterium*, *Shewanella* and *Aeromonadaceae*. As expected, 60% CO_2_ appeared to inhibit growth of a wide range of bacteria naturally present on cod fillets, including *Shewanella* and *Aeromonadaceae*, but to a less degree *Photobacterium* and *Carnobacterium/Carnobacteriaceae*, which dominated at the end of storage. In O_2_/CO_2_ atmosphere, there was a rapid growth and dominance *of Carnobacterium/Carnobacteriaceae*. This was not seen by Hovda et al. [24], either for the CO_2_/N_2_ or the CO_2_/O_2_ atmospheres, although they detected *Carnobacterium* at Day 0. However, Antunes-Rohling et al. [29] found that *Carnobacterium* can contribute to spoilage of hake, in addition to *Photobacterium* under CO_2_/N_2_ atmosphere.

**Figure 4:**
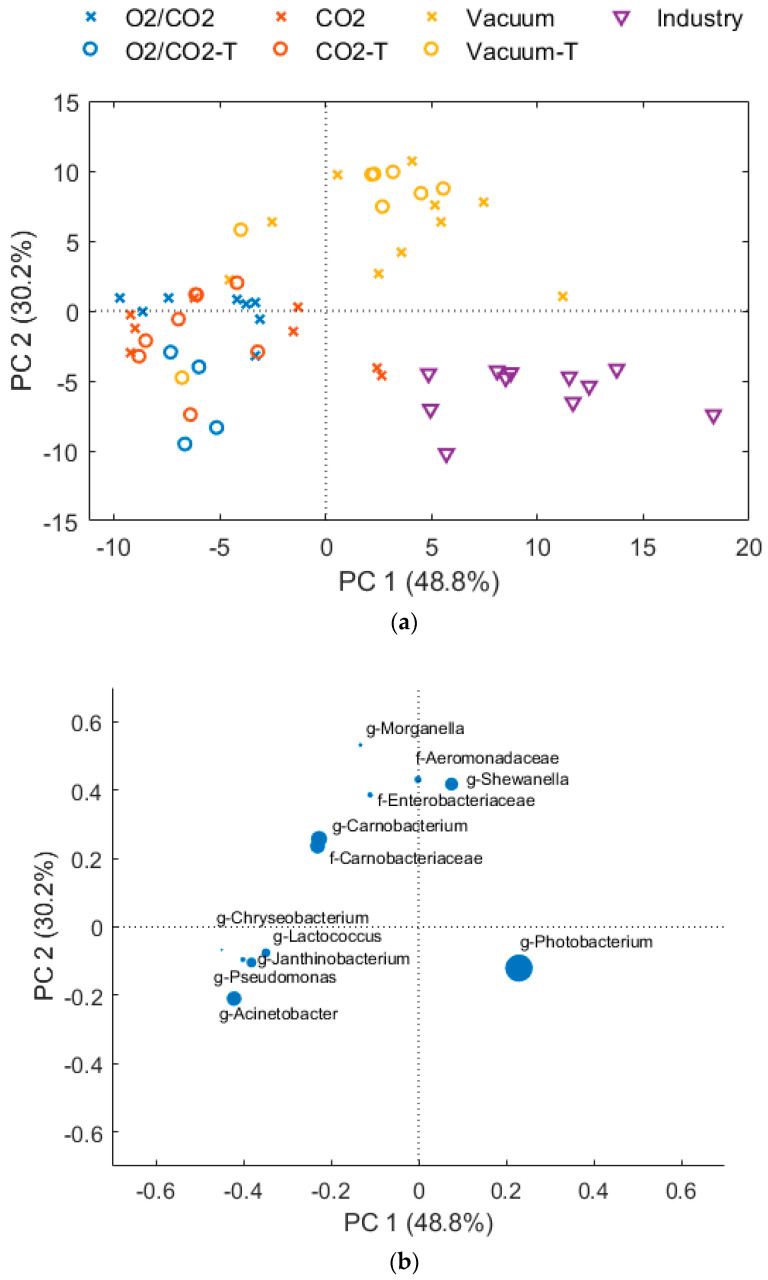
PCA of bacteriota after 1, 5, 8, 13 and 15 days of storage of fresh (cross symbol) and thawed (circle symbol) samples. The “CO_2_” samples (red color) are packaged with 60% CO_2_, 40% N_2_ and a CO_2_ emitter, and “O_2_/CO_2_” (blue color) is packaged with 60% CO_2_ and 40% O_2_. “Industry” samples (purple triangle symbol) and “Vacuum” samples (yellow color) are packaged with vacuum. (**a**) Scores plot, days of storage are indicated by label, (**b**) loading plot, dot size corresponds to relative abundance.

The bacterial growth in vacuum packaged cod fillets that were directly delivered from a fishing boat to the fish factory at the same day (termed “Industry”), differed significantly from the fillets produced from live stored cod (Figure 2, Figure 3 and Figure 4). No lag phase and a rapid growth of *Photobacterium* up to almost 8 log10 CFU/g after 13 days was observed (7.5 log10 CFU/g after 8 days). The origin of the fillets (wild caught vs live stored with a different initial contamination and/or fish flesh properties) and time of processing (later in the day) could have been the cause of the large differences. 

Overall, the results were in accordance with previous literature that identify a shift in the dominant populations during storage of cod as an effect of storage conditions [8,10]. We have also in an earlier study found a dominance of *Carnobacterium* during storage of 40% O_2_/60% CO_2_ packaged cod [23]. However, Rudi et al. [8] detected a strong association of coalfish with *Photobacterium*, but salmon was associated with *Carnobacterium* and *Brochothrix* (both species packaged with CO_2_/N_2_). Similar to the present study, Hovda et al. [24] did not find any difference in total bacterial count between cod packed in CO_2_ with and without high-O_2_. However, in contrast to our findings, *Pseudomonas* dominated, not *Carnobacterium* in cod packaged with O_2_/CO_2_ gas. Another study has reported lower bacterial growth rates for cod packaged with high-O_2_/CO_2_ compared to CO_2_/N_2_ [28]. Differences between studies may reflect differences between raw materials used and the initial contamination, as shown in the present study where both counts and composition of the contamination of fresh cod varied significantly between three factories. Difference in bacterial composition between different batches is also reported on hake [29]. 

#### 3.3.2. The Effect of Short-Term Freezing on Development of the Bacteriota during Refrigerated Storage 

Depending on packaging gas, the short-term freezing and thawing affected both the total growth rate (Figure 2 and Table A1) and the bacteriota (Supplementary Table S1 and Figure 3 and Figure 4) (*p* = 0.002 and *p* < 0.001, respectively). Overall, it seemed like the freezing process eliminated or strongly reduced the relative abundance of *Photobacterium* in all packaging types until the end of storage and increased the relative abundance of *Pseudomonas* and *Acinetobacter* early in the storage period. For thawed, vacuum packed cod, a rapid growth of *Shewanella* and *Carnobacterium/Carnobacteriaceae* were observed, resulting nearly 8 log10 CFU/g numbers after 15 days of storage. As also found for fresh samples, CO_2_ inhibited *Shewanella*, and when *Photobacterium* is reduced by freezing/thawing *Carnobacterium/Carnobacteriaceae* dominated. Moreover, in cod packaged with O_2_/CO_2,_ there was a rapid growth of *Carnobacterium/Carnobacteriaceae*, accompanied by *Acinetobacter* and *Pseudomonas* in the absence of *Photobacterium* (for details see Supplementary Table S1). A similar effect of freezing on the microbial composition has also been reported by Emborg et al. [73] and Bøknæs et al. [33]. The present study and other studies imply that important spoilage bacteria, such as *Photobacterium*, *Shewanella* and *Pseudomonas* can be inhibited by a combination of packaging gas and freezing-thawing processes. In the present study, they were replaced by *Carnobacterium/Carnobacteriaceae*. *Carnobacterium* has been detected as the prevalent bacteria in tuna, shrimps, swordfish, and cod packaged in high-oxygen MAP (80% O_2_, 20% N_2_, and 60% CO_2_, 40% O_2_) [16,74]. It has been reported that *Carnobacterium* is less competitive compared to *Photobacterium* under CO_2_/N_2_ atmosphere, but grows well together with *Shewanella baltica* [67,75,76].

Although the effects of CO_2_ and O_2_ on the bacteriota from the experiment with cod fillets were partly in agreement with the model experiment with agar plates, it was obvious that much less inhibitory effect were found with a food matrix and that growth rates in cod were different from the cod based nutrient agar. For example, *Photobacterium* showed rapid growth in cod, but very slow growth on agar. There are probably several reasons for this, such as different strains, mixed bacterial communities, better exposure to the gas, and differences between nutrients between the two systems. However, growth on fish is the most relevant as weakness of using model systems may occur.

#### 3.3.3. Effect of Packaging Atmospheres and Short-Term Freezing on Sensory Profiles and Volatile Compounds after Storage 

**Figure 5:**
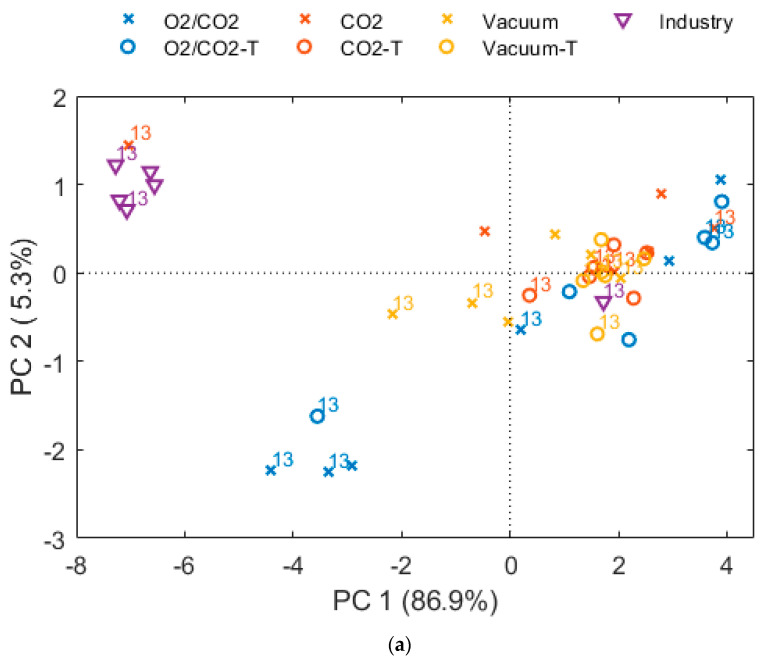

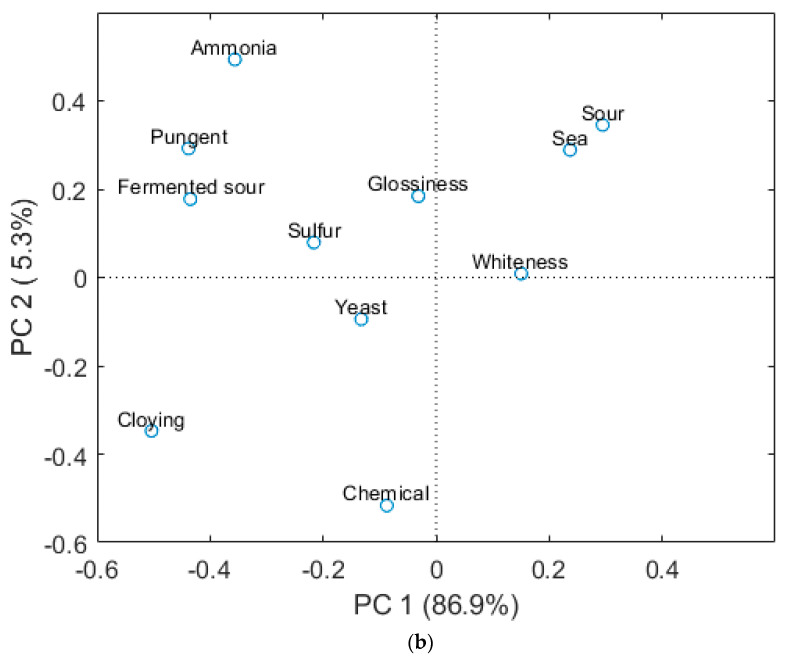
PCA analysis of sensory attributes evaluated after 8 days and 13 days of storage for the fresh (cross symbol) and thawed (circle symbol) samples. The “CO_2_” samples (red color) are packaged with 60% CO_2_, 40% N_2_ and a CO_2_ emitter, and “O_2_/CO_2_” (blue color) is packaged with 60% CO_2_ and 40% O_2_. “Industry” samples (purple triangle symbol) and “Vacuum” samples (yellow color) are packaged with vacuum. (**a**) Scores plot (13 days of storage are indicated by label), (**b**) loading plot: odor attributes.

**Figure 6:**
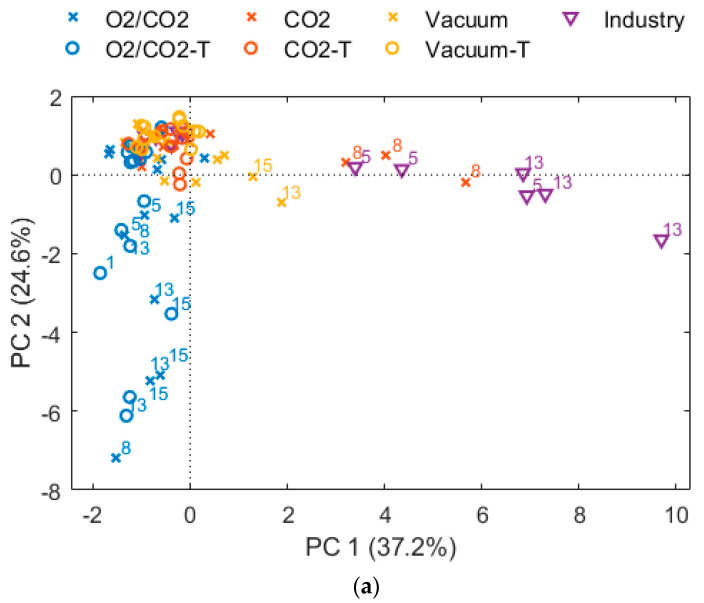

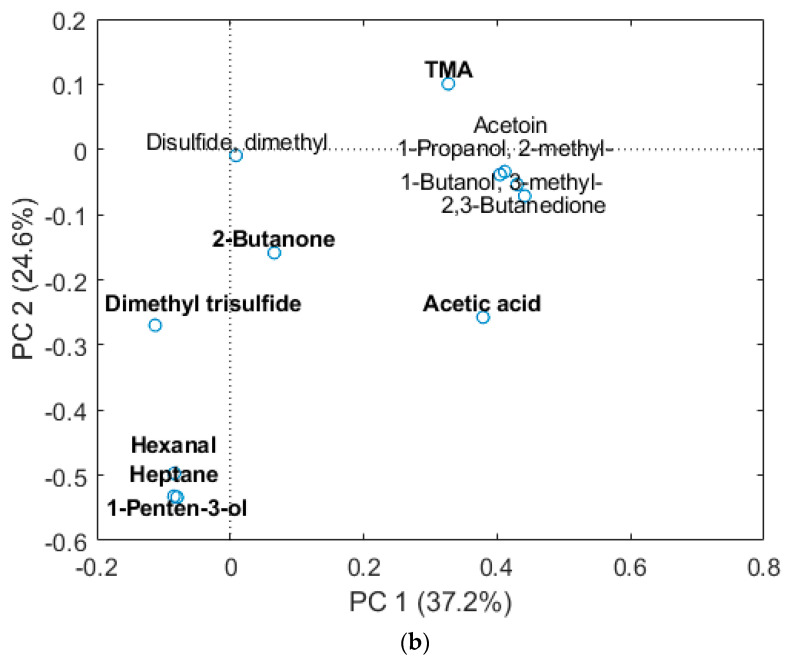
PCA of volatile components after 8 and 13 days of storage for fresh (cross symbol) and thawed (circle symbol) samples, Table 2. samples (red color) are packaged with 60% CO_2_, 40% N_2_ and a CO_2_ emitter, and “O_2_/CO_2_” (blue color) is packaged with 60% CO_2_ and 40% O_2_. “Industry” samples (purple triangle symbol) and “Vacuum” samples (yellow color) are packaged with vacuum. (**a**) Score plot and (**b**) loading plot. Days of storage are indicated by label for those with score PC1 > 1 or score PC2 < 1. Loading plot: Compounds with significant effect of packaging gas (FDR < 0.05) from the 5050 manova/rotation test for design samples are in bold letters.

The different packaging concepts, both the type of packaging gas and whether the cod had been frozen and thawed, affected the sensory profiles and level of metabolites (Table A5 and Table A6, Figure 5 and Figure 6). Nevertheless, the “Industry” fillets were deviant compared to the other ones with higher intensity scores of the attributes cloying, fermented sour, ammonia, and pungent compared to the other packaging variants (Table 4 and Figure 5). These attributes can be related to the presence and metabolism of *Photobacterium*, as this bacterium totally dominated during the refrigerated storage. 

In the fresh cod fillets of “CO_2_” and “Vacuum” the bacteriota was dominated by *Photobacterium*, and *Shewanella* and *Photobacterium*, respectively. The bacterial levels were probably too low (<7 log10 CFU/g) to cause significant sensory changes, especially for the level of *Shewanella* (sensory impact at levels above 8 log10 CFU/g, according to Dalgaard et al. [39]). In general, the intensities of negatively associated sensory attributes were low after 13 days for all cod fillets packaged in CO_2_ or vacuum (Table 4). Although *Photobacterium* dominated in CO_2_ packaged fillets, it seemed to have a slower growth compared to what was found for the vacuum packaged fillets. A difference in odor intensity scores between vacuum and CO_2_ packaged could possibly been detected if a further time of storage had been conducted, as differences in odor attributes is previously found between similar packaging methods for cod fillets [12]. 

After 13 days of storage, fresh cod fillets packed in O_2_/CO_2_ showed significantly higher intensities for cloying and chemical odor compared to fillets packaged with “CO_2_” or in vacuum. The difference in bacteriota for the fresh samples were clear; O_2_/CO_2_ samples were dominated by *Carnobacterium/Carnobacteriaceae* whereas all other samples were dominated by *Photobacterium* and *Photobacterium/Shewanella*. Higher intensities of cloying and chemical odor may therefore be caused by *Carnobacterium* or other members of *Carnobacteriaceae*, however this must be investigated further both to identify the specific species and to show any causal relationship.

The thawed fillets were dominated by *Acinetobacter* and *Carnobacterium*. The growth of *Acinetobacter* was in contradiction to what was found in the screening test, which showed almost completed inhibition of *Acinetobacter* under different modified atmospheres. *Acinetobacter* has not been reported as a spoilage bacterium and seems to be outcompeted by *Carnobacterium* during further storage (Figure 2).

Laursen et al. [77] reported development of off-flavors like chlorine, chemical, malty, and sour with growth of *Carnobacterium* on chilled MAP Nordic shrimps. The attribute chlorine was not included in the sensory attribute list in our study but was a part of the description of the attribute chemical (Table A3). Additionally, chlorine was mentioned by the sensory panelist for O_2_/CO_2_ packaged fillets, but also for fillets packed with vacuum and CO_2_. At the same time, the presence of 1-penten-3-ol, heptane, and hexanal were significant effected by packaging gas with higher levels for the cod packaged with O_2_/CO_2_ (Figure 6). These components are associated with oxidation of lipids facilitated by the high-oxygen gas mixture [23,78]. The chemical attribute might therefore also be related to oxidation of the fillets, but probably not very likely because of the low lipid content in cod fillet, though this must be further investigated. 

Samples that had gone through the freezing/thawing process had lower intensities of fermented sour, and higher sour odor and sea odor after 13 days storage (Table 4). The effect of freezing/thawing depended on packaging gas, and significant interactions between the freezing thawing process and packaging gas was observed for ammonia, cloying, glossiness, pungent, and fermented sour odor (Table A5). Results therefore indicate that freezing/thawing may have a positive effect on the product quality, which correspond to findings by Guldager et al. [32]. 

Several volatile components of alcohols, esters, ketones, aldehydes, and acids were detected, with a selection of the relevant components shown in Figure 6 and Table A6. TMA is commonly known as a bacterial metabolite of *Photobacterium* [7,38,39], with an estimation of 30 times higher TMA production by *Photobacterium* compared to *Shewanella* [39]. Cod fillets dominated by *Carnobacterium* have not shown any development of TMA as demonstrated on fillets dominated by *Photobacterium* [23]. In addition, an association between acetic acid and *Photobacterium* has been reported [79]. Our results showed that the levels of TMA and acetic acid were affected by packaging gas and process, in addition to 2-butanone and dimethyl trisulfide that were also affected by packaging (Table A6), and as shown in Figure 6. The contents of TMA and acetic acid were also especially pronounced for ordinary industrial vacuum packaged cod fillets (“Industry”) and some of the fillets packaged with CO_2_ that were dominated by *Photobacterium* (Figure 6). After eight days of storage, the “Industry” samples had reached high bacterial levels (7.5 log10 CFU/g) and a higher intensity of pungent, cloying, sour fermented, ammonia, and sulfur compared to the other samples (data not shown). A more pronounced difference in the quality of the different packaging concepts would probably have been detected if sensory analyses had been performed after the 15 days of storage.

## 4. Conclusions

A number of strategies have been proposed to extend the shelf life of cod, such as strict production hygiene, optimization of packaging gases, long-term and short-term freezing and thawing, and extreme cooling regimes. The present work illustrates how technologies that have been reported to inhibit or reduce known spoilage bacteria may not always extend the shelf life. For example, the bacterial growth under vacuum packaging and aerobic storage proved similar for fresh cod. However, combined with freezing/thawing, this packaging technology may improve sensory quality due to a shift in dominating bacteriota from *Photobacterium* (high spoilage potential) to *Shewanella*/*Carnobacterium*. 

The present study supports previous studies showing that storage under different modified atmospheres (CO_2_ and N_2_ or O_2_) can be used to control the dominating bacteriota, including specific spoilage organisms, such as *Shewanella*, *Photobacterium*, and *Pseudomonas*. However, when these bacteria were inhibited, a rapid growth of *Carnobacterium* was found. The dominance of *Carnobacterium* was linked to the negatively associated attributes of cloying and chemical odor, and possibly also chlorine odor. *Carnobacterium* spp. may thereby be defined as a specific spoilage organism in cod when other spoilage bacteria are reduced or inhibited. 

Altogether, this study presents results that are important to achieve improved quality control of fish fillet products and ultimately inhibit food waste in the value chain.

## Figures and Tables

**Table 1:** Bacteria (%) identified (by 16S rRNA gene sequencing of colonies) from three different processing plants, one day after slaughtering and filleting. At Plant 1 samples were taken from both the skin and from the flesh (fillet). Plant 2 and 3 were industrial processing plants with sampling of the fillets.

	Plant 1		Plant 2	Plant 3	Colonies (*n*),
Colonies of Bacterial Genus	Filet (*n* = 6)	Skin (*n* = 6)	Filet (*n* = 5)	Filet (*n* = 5)	Filet/Skin
*Photobacterium*	92.6	78.9		3.8	88/30
*Pseudomonas*	1.1	7.9	27.8	30.8	14/3
*Shewanella*			11.1		2/0
*Aeromonas*			5.6		1/0
*Psychrobacter*	1.1	5.3	11.1	19.2	8/2
*Arthrobacter*		2.6		26.9	7/1
*Acinetobacter*			22.2	3.8	5/0
*Janthinobacterium*	3.2				3/0
*Chryseobacterium*		2.6		3.8	1/1
*Stenotrophomonas*	1.1				1/0
*Sphingobacterium*			5.6	3.8	2/0
*Flavobacterium*			5.6	7.7	3/0
*Massilia*	1.1	2.6			1 /1
*Comamonas*			5.6		1/0
*Myroides*			5.6		1/0
Colonies in sample (*n*)	94	38	18	26	Total: 138/38
Total count, log10 CFU/g	1.2 ± 1.0	2.0 ± 0.8	4.7 ± 0.1	3.6 ± 0.3	

**Table 2:** Bacteria (%) characterized (by 16S rRNA gene sequencing of colonies) from the same samples of fish fillets (*n*) after 6 (industrial processing plants) and 9 days (controlled facility) of storage (3 °C).

	Plant 1, Day 9		Plant 1, Day 9		Plant 2, Day 6	Plant 3, Day 6	
Colonies of Bacterial Genus	Air (*n* = 4)	Air (Thawed) (*n* = 4)	Vacuum (*n* = 4)	Vacuum (Thawed) (*n* = 4)	Air (*n* = 3)	Air (*n* = 3)	Colonies (*n*)
*Photobacterium*	91.7		98.1			23.8	101
*Pseudomonas*	8.3	98.0	1.9	85.4	39.1	71.4	113
*Shewanella*					30.4	4.8	8
*Aeromonas*					4.3		1
*Psychrobacter*				9.8			4
*Acinetobacter*					17.4		4
*Myroides*					4.3		1
*Pseudochrobactrum*					4.3		1
*Carnobacterium*		2.0		4.9			
Colonies in sample (*n*)	48	50	53	41	21	21	Total: 236
Total count, log10 CFU/g	6.3 ± 1.0	4.2 ± 0.8	5.7 ± 0.5	4.5 ± 1.2	6.3 ± 0.6	6.8 ± 0.5	

**Table 3:** Growth screening of bacterial strains. Two–five strains (N) per genus were tested. Logarithmic change in bacterial numbers after 21 days of 4 °C storage on cod agar trays inoculated with selected bacteria strains under different atmospheric conditions. Mean values for 2–5 strains for each genus (from Table A1) are given. Positive numbers indicate growth. For each row, entries with the same letter attached to them are not significantly different at the 0.05 level.

Genus	Air	100% N_2_	40% CO_2_, 60% N_2_	60% CO_2_, 40% N_2_	60% O_2_, 40% CO_2_	40% O_2_, 60% CO_2_	N (Number of Strains)
*Carnobacterium*	6.7 ^a^	6.7 ^a^	5.1 ^b^	4.3 ^b^	5.0 ^c^	3.7 ^d^	2
*Pseudomonas*	7.9 ^a^	7.6 ^a^	2.7 ^b^	1.5 ^c^	1.3 ^c^	0.6 ^c^	5
*Aeromonas*	7.6 ^a^	7.5 ^a^	2.1 ^b^	2.2 ^b^	0.4 ^c^	0.3 ^c^	3
*Shewanella*	7.5 ^a^	6.5 ^a^	3.9 ^b^	2.0 ^c^	0.1 ^d^	0.1 ^d^	4
*Acinetobacter*	6.4 ^a^	3.1 ^a,b^	1.8 ^a,b^	0.3 ^b^	0.4 ^b^	0.0 ^b^	2
*Photobacterium*	3.5 ^a^	3.8 ^a^	2.2 ^a^	2.5 ^a^	−1.3 ^b^	−1.3 ^b^	2

**Table 4:** Sensory scores of odor attributes (1 = low intensity, 9 = high intensity) after 13 days of storage (2 °C). For each column, entries with the same letter attached to them are not significantly different at the 0.05 level.

	Sour Odor ^1^	Sea Odor ^1^	Cloying	Fermented Sour	Yeast	Chemi-Cal	Sulfur	Ammonia	Pungent	White-Ness	Glossiness
CO2	2.78 ^a^	2.44 ^ab^	3.71 ^b^	3.26 ^bcd^	1.49 ^abc^	1.72 ^b^	2.40 ^ab^	2.96 ^ab^	3.01 ^b^	5.59 ^ab^	4.85 ^a^
CO_2_-T	3.02 ^a^	2.75 ^a^	3.17 ^b^	2.32 ^cd^	1.22 ^c^	1.75 ^b^	2.74 ^ab^	2.22 ^b^	1.92 ^bc^	5.58 ^ab^	3.35 ^b^
Vacuum	2.09 ^ab^	2.17 ^abc^	3.64 ^b^	3.51 ^bc^	1.32 ^c^	1.96 ^b^	2.55 ^ab^	2.65 ^b^	2.04 ^bc^	5.39 ^b^	3.99 ^ab^
Vacuum-T	2.98 ^a^	2.77 ^a^	3.07 ^b^	2.66 ^bcd^	1.14 ^c^	1.99 ^b^	1.85 ^b^	1.79 ^b^	1.57 ^c^	6.10 ^a^	4.61 ^a^
O_2_/CO_2_	1.55 ^b^	1.37 ^c^	5.71 ^a^	3.91 ^ab^	2.06 ^a^	3.10 ^a^	2.62 ^ab^	2.58 ^b^	3.06 ^b^	5.16 ^b^	4.15 ^ab^
O_2_/CO_2_-T	3.10 ^a^	2.63 ^a^	3.12 ^b^	2.22 ^d^	1.36 ^bc^	2.22 ^ab^	2.29 ^b^	1.89 ^b^	2.23 ^bc^	6.07 ^a^	4.15 ^ab^
Industry	1.68 ^b^	1.61 ^bc^	5.90 ^a^	4.84 ^a^	1.99 ^ab^	1.84 ^b^	3.43 ^a^	4.27 ^a^	4.54 ^a^	5.42 ^b^	4.42 ^a^

^1^ positive associated odor attributes; Green color means that scores are significant different to “Industry” (*p* = 0.05), and orange color means score different to both “Industry” and the other packaging methods.

## Data Availability

The data presented in this study are available on request to the corresponding author.

## Supplementary Materials

The following are available online at https://www.mdpi.com/article/10.3390/foods10081754/s1, Table S1: Bacteriota relative abundance table (Excel file).

## Appendix A

**Table A1:** Bacterial strains (20) used in growth screening on cod agar plates at different packaging gas mixtures. The bacterial strains were selected from the fillets from Plant 2 and 3 (aerobically stored fillets), which were the commercial processing plants, but similar strains were also detected from Plant 1. Two strains were from vacuum packaged fillets of Plant 1. Three strains were chosen from former industrial studies (*).

Bacterial Taxa Bacterial Taxa	Source	Strain Number
*Pseudomonas putida*	Plant 2, Day 1	MF 7459
*Pseudomonas syringae*	Plant 2, Day 6	MF 7464
*Pseudomonas psychrophila*	Plant 2, Day 6	MF 7465
*Pseudomonas fluorescens*	Plant 3, Day 6	MF 7470
*Pseudomonas fragi*	Plant 3, Day 6	MF 7471
*Photobacterium phosphoreum*	Plant 3, Day 1	MF 7469
*Photobacterium* sp.	Plant 3, Day 6	MF 7473
*Shewanella* sp.	Plant 2, Day 1	MF 7460
*Shewanella* sp.	Plant 3, Day 6	MF 7472
*Shewanella baltica*	From salmon-industry *	MF 5509
*Shewanella putrefaciens*	From salmon-industry *	MF 5496
*Aeromonas* sp.	Plant 2, Day 1	MF 7461
*Aeromonas rivuli*	Plant 2, Day 6	MF 7466
*Aeromonas salmonicida*	From salmon-industry *	MF 5413
*Acinetobacter* sp.	Plant 2, Day 1	MF 7462
*Acinetobacter* sp.	Plant 2, Day 6	MF 7463
*Arthrobacter* sp.	Plant 3, Day 1	MF 7467
*Psychrobacter glacincola*	Plant 3, Day 1	MF 7468
*Carnobacterium* sp.	Plant 1, Day 9 (vacuum, thawed)	MF 7458
*Carnobacterium maltaromaticum*	Plant 1, Day 9 (vacuum, thawed)	MF 7457

**Table A2:** The different packaging gas mixtures used for packaging of cod agar plates for growth screening, and the measured levels (%) of CO_2_ and O_2_ after 13 days of refrigerated 4 °C storage of at total storage period of three weeks.

Premix Packaging Gas	Gas Measured Day 13
60% CO_2_, 40% N_2_	53.1 ± 0.5% CO_2_, 0.5 ± 0.3% O_2_
40% CO_2_, 60% N2	34.8 ± 0.4% CO_2_, 0.3 ± 0.4% O_2_
60% CO_2_, 40% O_2_	51.4 ± 0.2% CO_2_, 44.9 ± 0.2% O_2_
40% CO_2_, 60% O_2_	32.3 ± 0.0% CO_2_, 63.7 ± 0.0% O_2_
100% N_2_	1.0 ± 0.2% CO_2_, 0.02 ± 0.0% O_2_
Air	0.3 ± 0.1% CO_2_, 20.3 ± 0.0% O_2_

**Table A3:** Sensory vocabulary for raw samples of cod fillets (*Gadus morhua*) of odor attributes and for color/appearance. No intensity = no presence of the specific attributes, evident intensity = evident presence of the specific attributes.

Sensory Attributes	Description of Attribute
Odor	
Seawater	Fresh salty, sea, ocean
Sour	Related to a fresh, balanced odor due to the presence of organic acids
Fermented sour	Fermented sour odor, tainted
Cloying	An unfresh and sickening odor
Yeast	Odor of yeast
Chemical	Related to odors like diesel, burnt, scorched, rubber, chlorine
Sulfur	Sulfur odor, boiled egg, cabbage, onion
Ammonia	odor of ammonia
Pungent	Relates to a sharp and stinging odor
Color/Appearance	
Whiteness	Paleness, lack of pure color or black
Glossiness	overall impression

**Table A4:** 5050 manova results for bacteriota. The table shows the false discovery rate (FDR) adjusted *p*-values and the explained variance for each of the terms included in the model. The "*" is showing the interactions.

OTUlabel	Day	Gas	Process	Day * Gas	Day * Process	Gas * Process
*Explained variance (%)*	13.4	50.1	4.3	3.2	1.5	4.9
*g-Chryseobacterium*	0.000	0.000	0.090	0.968	0.654	0.468
*f-Carnobacteriaceae; Other*	0.054	0.001	0.889	0.461	0.654	0.009
*g-Carnobacterium*	0.066	0.002	0.316	0.461	0.654	0.031
*g-Lactococcus*	0.028	0.000	0.346	0.671	0.654	0.070
*g-Janthinobacterium*	0.000	0.000	0.000	0.968	0.654	0.666
*f-Aeromonadaceae*	0.099	0.000	0.501	0.461	0.654	0.070
*g-Shewanella*	0.230	0.000	0.289	0.597	0.654	0.024
*f-Enterobacteriaceae*	0.054	0.000	0.316	0.461	0.654	0.439
*g-Morganella*	0.256	0.000	0.056	0.461	0.483	0.024
*g-Acinetobacter*	0.000	0.000	0.003	0.461	0.654	0.148
*g-Pseudomonas*	0.000	0.000	0.001	0.671	0.882	0.666
*g-Photobacterium*	0.401	0.002	0.021	0.540	0.745	0.148

**Table A5:** ANOVA of sensory descriptive analysis on odors for packaging concepts in storage experiment without industrial samples. Mixed model with effect of assessor, gas, process (freezing/thawing), replicate and two-way interactions. Assessor and interactions with assessors are random effects, other fixed. The "*" is showing the interactions.

Attribute	Gas	Process	Interaction	Replicate	Gas * Replicate	Process * Replicate
Ammonia odor	0.015	0.000	0.009	0.005	0.000	0.000
Cloying odor	0.059	0.000	0.004	0.148	0.000	0.000
Yeast odor	0.044	0.000	0.144	0.715	0.000	0.061
Chemical odor	0.063	0.091	0.147	0.157	0.100	0.086
Seawater odor	0.188	0.001	0.230	0.821	0.000	0.000
Pungent odor	0.004	0.000	0.000	0.105	0.000	0.000
Fermented sour odor	0.356	0.000	0.010	0.036	0.000	0.000
Sulfur odor	0.088	0.010	0.610	0.257	0.000	0.000
Sour odor	0.669	0.000	0.217	0.642	0.000	0.000
Glossiness	0.619	0.136	0.000	0.734	0.130	0.000
Whiteness	0.292	0.000	0.105	0.003	0.000	0.002

**Table A6:** 5050 manova results for selected volatile organic compounds. The table shows the false discovery rate (FDR) adjusted *p*-values for each of the terms included in the model. The "*" is showing the interactions.

Compound	Day	Gas	Process	Day * Gas	Day * Process	Gas * Process
Explained variance (%)	18.7	14.7	2.7	12.1	3.7	3.1
Trimethylamine	0.0001	0.0001	0.0421	0.00165	0.63499	0.86033
Dimethyl disulfide	0.29786	0.52467	0.13338	0.10174	0.39613	0.0776
Dimethyl trisulfide	0.47753	0.02586	0.40159	0.89936	0.59946	0.1146
3-methyl-1-Butanol	0.0001	0.1159	0.12875	0.0001	0.63499	0.1091
2-methyl-1-Propanol	0.03457	0.40634	0.10637	0.00233	0.30765	0.1091
1-Penten-3-ol	0.66168	0.0001	0.83773	0.97357	0.39613	0.96596
Heptane	0.00887	0.00017	0.83773	0.89936	0.65986	0.57603
Hexanal	0.29786	0.00025	0.96558	0.30384	0.6959	0.99812
2-Butanone	0.0001	0.03025	0.37694	0.11575	0.60465	0.7585
2,3-Butanedione	0.0001	0.59535	0.44496	0.30384	0.65986	0.86033
Acetoin	0.0001	0.64372	0.21098	0.00347	0.63499	0.57603
Acetic acid	0.04599	0.04577	0.0077	0.27464	0.2337	0.95961
